# *Vibrio cholerae* in rural and urban Bangladesh, findings from hospital-based surveillance, 2000–2021

**DOI:** 10.1038/s41598-023-33576-3

**Published:** 2023-04-19

**Authors:** Rina Das, Sabiha Nasrin, Parag Palit, Rukaeya Amin Sobi, Al-Afroza Sultana, Soroar Hossain Khan, Md. Ahshanul Haque, Sharika Nuzhat, Tahmeed Ahmed, A. S. G. Faruque, Mohammod Jobayer Chisti

**Affiliations:** 1 Nutrition and Clinical Services Division, International Centre for Diarrheal Disease Research, Bangladesh (icddr, b), 68 Shaheed Tajuddin Ahmed Sharani, Dhaka, 1212 Bangladesh; 2 Gangarosa Department of Environmental Health, Rollins School of Public Health, Emory University, Atlanta, GA 30322 USA; 3 Department of Biostatistics and Epidemiology, School of Public Health and Health Sciences, University of Massachusetts, Amherst, MA 01003 USA; 4 University of Virginia School of Medicine, Charlottesville, VA USA; 5 James P. Grant School of Public Health, BRAC University, Dhaka, 1212 Bangladesh; 6 Department of Global Health, University of Washington, Seattle, WA 98104 USA

**Keywords:** Microbiology, Diseases, Gastroenterology, Risk factors

## Abstract

With more than 100,000 cases estimated each year, Bangladesh is one of the countries with the highest number of people at risk for cholera. Moreover, Bangladesh is formulating a countrywide cholera-control plan to satisfy the GTFCC (The Global Task Force on Cholera Control) Roadmap's goals. With a particular focus on cholera trends, variance in baseline and clinical characteristics of cholera cases, and trends in antibiotic susceptibility among clinical isolates of *Vibrio cholerae,* we used data from facility-based surveillance systems from icddr,b’s Dhaka, and Matlab Hospitals from years 2000 to 2021. Female patients comprised 3,553 (43%) in urban and 1,099 (51.6%) in rural sites. Of the cases and most patients 5,236 (63.7%) in urban and 1,208 (56.7%) in the rural site were aged 15 years and more. More than 50% of the families belonged to the poor and lower-middle-class; in 2009 (24.4%) were in urban and in 1,791 (84.2%) were in rural sites. In the urban site, 2,446 (30%) of households used untreated drinking water, and 702 (9%) of families disposed of waste in their courtyard. In the multiple logistic regression analysis, the risk of cholera has significantly increased due to waste disposal in the courtyard and the boiling of water has a protective effect against cholera. Rotavirus (9.7%) was the most prevalent co-pathogen among the under-5 children in both sites. In urban sites, the percentage of *V. cholerae* along with co-existing ETEC and *Campylobacter* is changing in the last 20 years; *Campylobacter* (8.36%) and Enterotoxigenic *Escherichia coli* (ETEC) (7.15%) were the second and third most prevalent co-pathogens. *Shigella* (1.64%) was the second most common co-pathogen in the rural site. Azithromycin susceptibility increased slowly from 265 (8%) in 2006–2010 to 1485 (47.8%) in 2016–2021, and erythromycin susceptibility dropped substantially over 20 years period from 2,155 (98.4%) to 21 (0.9%). Tetracycline susceptibility decreased in the urban site from 2051 (45.9%) to 186 (4.2%) and ciprofloxacin susceptibility decreased from 2,581 (31.6%) to 1,360 (16.6%) until 2015, then increased 1,009 (22.6%) and 1,490 (18.2%) in 2016–2021, respectively. Since 2016, doxycycline showed 902 (100%) susceptibility. Clinicians need access to up-to-date information on antimicrobial susceptibility for treating hospitalized patients. To achieve the WHO-backed objective of eliminating cholera by 2030, the health systems need to be put under a proper surveillance system that may help to improve water and sanitation practices and deploy oral cholera vaccines strategically.

## Introduction

*Vibrio cholerae* is a gram-negative, motile, curved, or comma-shaped rod that is facultatively anaerobic, and aquatic in nature that causes cholera, an acute diarrheal illness spread by the fecal–oral route^1^, but the environment also plays an important role^1^. Only two serogroups of *V. cholerae*, O1, and O139, are considered causative agents of epidemic cholera^2^. *V. cholerae* O1 has two biotypes, classical and El Tor. Each biotype has three serotypes: Ogawa, Inaba, and Hikojima. *V. cholerae* O1 Hikojima is an unstable form and rarely occurs in nature^2,3^. It is self-evident that providing safe drinking water, proper sanitation, and maintaining good personal and food hygiene will limit the spread of cholera^1^. Developed countries around the world have accomplished such measures that ensure the limiting imitation of cholera^1,4^. On the other hand, it is a severe public health issue in low- and middle-income countries (LMICs), with an estimated 100,000 deaths per year^4,5^.

Cholera is more common in regions with poor access to clean water and sanitation, such as those affected by conflict or natural disasters^1^. Millions have been afflicted by cholera pandemics since the early 1800s, with the seventh still underway since 1961^1,4,5^. Cholera is thought to have originated in the Ganges delta, which runs across India and Bangladesh making it one of the world's cholera hotspots^5^. Cholera is usually endemic in Bangladesh and spread by fecal–oral route but in terms of epidemics, the disease can spread rapidly in areas due to inadequate treatment of sewage and drinking water. However, in Bangladesh, the outbreak is ongoing and has no end in sight and transmission occurs consistently annually^6,7^. The impoverished population has a lack of access to clean drinking water, hygiene, and sanitation. In addition, the consequences of global warming, which are particularly prominent in Bangladesh are linked to an increased incidence of cholera^5^. Apart from this, several other factors are associated with the occurrence of cholera, including rainfall patterns, sea surface temperature, and the El Niño Southern Oscillation (EŇSO)^8^. Our data has the same seasonal outbreak pattern which is consistent with the seasonal outbreak of cholera in Dhaka and Matlab Hospital, icddr,b.

The International Centre for Diarrheal Disease Research, Bangladesh (icddr,b) runs two hospitals in Dhaka City that treats around 20,000 cholera patients each year (Surveillance data from Dhaka Hospital, icddr,b, 2018)^5^. Moreover, since 1966, we have had a unique opportunity to examine the epidemiological dynamics of cholera in Matlab, Bangladesh^5,9^, a rural research field site of icddr,b with a population of around 200,000 people (now 239,000), situated 30 miles southeast of Dhaka city close to the confluence of the Ganges and the Meghna rivers^5,10^. Owing to its persistently high cholera prevalence, Matlab has been one of the world's 'go-to' places for assessing cholera vaccines since the 1960s^3,5^. Both Whole Cell (WC) or Whole-Cell Monovalent (O1) Vaccine with Cholera Toxin B Subunit (WC-BS) both WC and WC-BS were evaluated in a large randomized, controlled field trial in Matlab, Bangladesh beginning in 1985^3,11,12^. Efficacy was assessed at 4-time points (6 months, 1 year, 3 years, and 5 years) over the 5-year follow-up period and reported for two age groups (2–5 years, > 5 years). In the third year, there was no evidence of any protection for children 2–5 years of age with either product^13^.

After three years, the vaccines provided no significant protection against any serotypes of cholera^10,14^. Cholera infection has been reported to provide solid protection against reinfection with a homologous strain for up to 3 years in many volunteer challenge studies^15,16^.

GTFCC roadmap focuses on preventing transmission in cholera hotspots through vaccines and better access to proper WASH facilities, to reduce cholera mortality by 90% and eliminate local transmission in at least 20 countries by 2030^1^. In LMICs, the situation regarding access to clean water and adequate sanitation is very critical. According to WHO/UNICEF estimates, more than two billion people are consuming water from fecally contaminated sources, and 2.4 billion people do not have access to basic sanitation facilities^17^. With more than 100,000 cases estimated each year, Bangladesh is one of the countries with the highest number of people at risk for cholera^4^. Moreover, Bangladesh is formulating a countrywide cholera-control plan to satisfy the GTFCC Roadmap's goals. Henceforth, understanding Bangladesh's cholera epidemiology remains integral for this purpose^18^. Evidence suggests that climate change and variability play a role in the emergence and reemergence of cholera^8^.

In this study, we present descriptive data from five-year intervals from 2000 to 2021, with a focus on cholera trends, variance in baseline and clinical characteristics of cholera cases, and trends in antibiotic susceptibility among clinical isolates of *V. cholerae* in urban and rural Bangladesh health facilities. Understanding the trend of cholera epidemics could help the health systems of Bangladesh prepare for better management as well as respond to possible outbreaks (e.g. by employing vaccines). As a result, it can aid in the development of preventative initiatives.

Data was collected from facility-based diarrheal disease surveillance systems (DDSS) in the urban Dhaka Hospital and the rural Matlab Hospital of icddr,b. The stool culture technique was used to isolate *V. cholerae* pathogen along with other co-pathogens detailed explanation is given below the in method section.

## Result

Between 2000 and 2021, there was a total of 10350 *V. cholerae* positive cases, with 8221 (79%) cases from the urban Dhaka Hospital and 2129 (20.6%) from the rural Matlab Hospital. Female patients made up 43% of the urban site and 51% of the rural site. In both urban and rural areas, the majority of patients were between the age of 15–60 years (59% and 47%, respectively) and more than 50% belonged to the poor and lower middle class (54.3%). 30% of households in the urban site did not treat their drinking water, specifically boiling, and 9% of families disposed of waste in their courtyard; but in the rural site 1.37% of the household treat drinking water and more than 99% households disposed their waste outside the courtyard. Dehydration levels were predominantly in the “some/severe” range at both sites, and most patients required both ORS and IV fluid 77.7% and 61.3% in urban and rural areas respectively (Supplementary table 1).

Table 1 shows the characteristics of *V. cholerae-*positive diarrhea patients admitted in urban and rural sites from 2000 to 2021, while 2000–2005 was compared with 2006–2010,2011–2015, and 2016–2021 admission years after adjusting for patient's age, sex, status of breastfeeding (under 3 children), use of antibiotic before hospitalization, number of family members, parental education, drinking water, toilet facility, water treatment method, garbage disposal method, asset index, and urban and rural sites. In all admission years, patients aged 15–60 years and above 60 years had a significantly higher likelihood of cholera and less than 3 days of diarrhea duration compared to the patients from the year 2000–2005. We found that the risk was significantly rising year by year. Compared to 2000–2005, the hazards were roughly two times as high in the years 2006–2010, three times as high in the years 2011–2015, and 3.5 times as high in the years 2016–2021. Boiling water was found to be protective, while waste disposal in their courtyard was associated with significantly increased cholera risk. When compared to the reference admission year, moderate to upper-class households were found to have a significantly higher risk of cholera. In contrast, patients were shown to have a lower likelihood of experiencing some dehydration from 2011 to 2021.

**Table 1:** Multiple logistic regression analysis results indicating characteristics of the *V. cholerae* positive diarrhea patients admitted in 2000–2005 while compared with 2006–2010, 2011–2015 and 2016–2021 admission years, in icddr,b Dhaka Hospital (urban) and Matlab Hospital (rural), Bangladesh.

Outcome variables	2006–2010		2011–2015		2016–2021	
	aOR (95% CI) *	*P* value	aOR (95% CI) *	*P* value	aOR (95% CI) *	*P* value
Age group						
< 5 years	Ref					
5–15 years	1.60(1.31, 1.96)	< 0.001	1.72(1.25,2.36)	0.001	1.65(1.20,2.28)	0.002
15–59 years	1.93(1.62, 2.29)	< 0.001	2.93(2.27, 3.79)	< 0.001	3.39(2.62, 4.39)	< 0.001
> 59 years	2.43(1.75, 3.36)	< 0.001	3.64(2.35, 5.65)	< 0.001	4.31(2.83, 6.58)	< 0.001
Sex (Female)	0.90(0.8, 1.03)	0.121	0.82(0.69, 0.98)	0.028	0.97(0.82, 1.14)	0.692
Duration of diarrhea before hospitalization						
> 3 days	Ref					
0–3 days	1.36 (1.01, 1.84)	0.046	2.31 (1.38, 3.89)	0.001	1.74 (1.09, 2.76)	0.018
Dehydration status						
No Dehydration	Ref					
Some Dehydration	0.86 (0.67, 1.09)	0.229	0.57 (0.42, 0.79)	0.001	0.56 (0.40, 0.78)	< 0.001
Severe Dehydration	1.09 (0.85, 1.39)	0.513	0.82 (0.59, 1.13)	0.229	0.93 (0.67, 1.28)	0.655
Rehydration fluid required						
Not required	Ref					
ORS only	0.44 (0.20, 0.96)	0.038	1.82 (0.38, 8.84)	0.457	0.40 (0.15, 1.05)	0.064
IV fluid only	1.02 (0.20, 5.22)	0.983	5.27 (0.54, 51.07)	0.151	15.94 (3.3, 76.92)	0.001
ORS and IV fluid	0.46 (0.21, 0.99)	0.047	1.71 (0.36, 8.26)	0.502	0.46 (0.18, 1.19)	0.109
Toilet facility						
Semi and non-Sanitary	Ref					
Sanitary	1.50 (0.84, 2.68)	0.166	2.91 (1.57, 5.41)	0.001	0.06 (0.01, 0.48)	0.008
Water treatment method						
No treatment	Ref					
Boil	0.77(0.64, 0.92)	0.004	0.77(0.6, 0.98)	0.033	0.61(0.49, 0.76)	< 0.001
Garbage disposal						
Outside	Ref					
Courtyard	5.53(3.25, 9.39)	< 0.001	11.73(6.74, 20.42)	< 0.001	12.81(7.48, 21.93)	< 0.001
Source of drinking water						
Tube well water	Ref					
Tape water and others	1.11 (0.93, 1.32)	0.235	1.02 (0.79, 1.31)	0.893	0.45 (0.36, 0.57)	< 0.001
Asset index						
Poor	Ref					
Lower middle	1.13(0.9, 1.41)	0.287	1.72(1.23, 2.39)	0.001	1.26(0.89, 1.79)	0.197
Middle	1.81(1.49, 2.2)	< 0.001	2.29(1.69, 3.11)	< 0.001	3.47(2.6, 4.63)	< 0.001
Upper middle	1.73(1.35, 2.22)	< 0.001	2.21(1.51, 3.22)	< 0.001	3.08(2.17, 4.38)	< 0.001
Rich	1.45(1.18, 1.79)	< 0.001	2.12(1.54, 2.92)	< 0.001	2.32(1.71, 3.16)	< 0.001
Co-Pathogens isolated						
*Shigella*	0.68(0.42, 1.09)	0.111	0.44(0.2, 0.98)	0.044	0.61(0.31, 1.21)	0.157
*Salmonella*	1.09(0.46, 2.57)	0.840	2.15(0.81, 5.72)	0.126	2.28(0.87, 5.98)	0.093
*Campylobacter*	2.23(1.57, 3.16)	< 0.001	5.01(3.38, 7.41)	< 0.001	8.74(6.09, 12.54)	< 0.001
*Aeromonas*	0.09(0.01, 1.14)	0.063	0.36(0.04, 3.62)	0.387	0.14(0.01, 1.59)	0.114
ETEC	3.80(2.47, 5.84)	< 0.001	7.90 (4.9, 12.74)	< 0.001	9.93(6.31, 15.61)	< 0.001
Site						
Urban	Ref					
Rural	0.90 (0.73, 1.11)	0.318	2.47(1.79, 3.42)	< 0.001	2.03(1.48, 2.79)	< 0.001

*Logistic regression was used on several outcome variables with the year of patient admission as exposure variables where the 2000–2005 group as the reference group and adjusted for patient's age, sex, the status of breastfeeding (under 3 children), use of antibiotic prior to hospitalization, number of family members, parental education, drinking water, toilet facility, water treatment method, garbage disposal method, asset index and urban & rural sites. Separate models were performed to see the association of characteristics of the *V. cholerae*-positive diarrhea patients admitted in 2000–2005 while compared with 2006–2010, 2011- 2015, and 2016–2021 admission years in both urban and rural facilities.

*aOR* adjusted odds ratio, *Ref.* Reference, ETEC. Enterotoxigenic *E.coli.*

**Figure 1:**
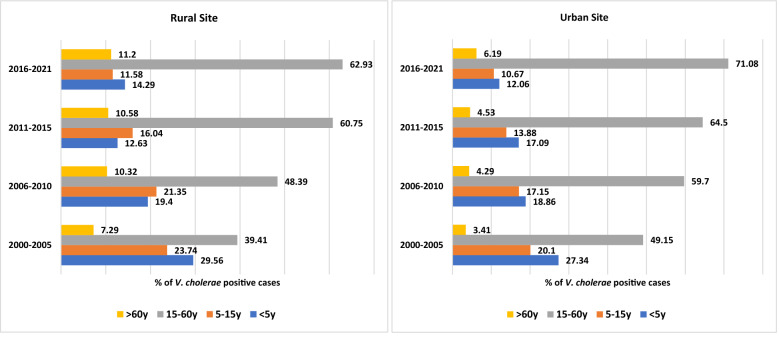
Age-specific distribution of *V. cholerae* positive patients admitted in the urban and rural sites during 2000–2021.

Figure 1 depicts the age-specific distribution of *V. cholerae* positive patients admitted in urban and rural settings from 2000 to 2021. The proportion of cases was always greater in those aged 15–60 years. The percentage of cholera infections increased gradually in this age group, from 39.41 to 62.93% in rural areas and from 49.15 to 71.08% in urban areas over the 20 years. However, the percentage of cholera infections decreased gradually in other age groups.

**Figure 2:**
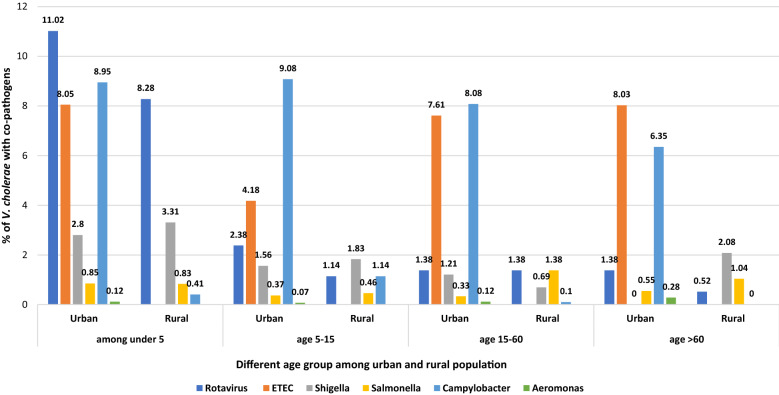
Co-pathogens isolated among *V. cholerae* positive patients admitted during 2000–2021.

Rotavirus (11.02% and 8.28%) was the most prevalent co-pathogen reported in both locations among the under-5 age group. The urban area had *Campylobacter* (8.36%) and Enterotoxigenic *Escherichia coli* (ETEC) (7.15%) as the second and third most frequently reported co-pathogens. *Shigella* (1.64%) was the second most common co-pathogen in the rural area among under 5 children, with a proportion that is almost identical to the urban site. ETEC and *Aeromonas* were not tested for rural samples (Fig. 2). *Campylobacter* was more common among under 5 (8.95%) and 5–15y age (9.08%) children.

**Figure 3:**
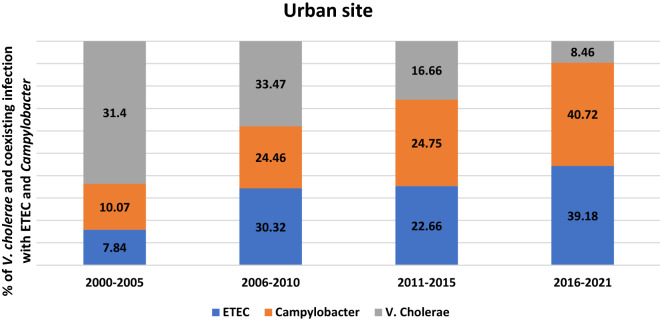
Percentage *V. cholerae* and co-existing infection *with* ETEC and *Campylobacter* in urban site.

The proportion of *Campylobacter* and ETEC co-infections rose over time in the urban site. Between 2000 and 2021, the prevalence of *Campylobacter* rose from 10.1 to 40.7% and ETEC rose from 7.8 to 39%. ETEC and *Aeromonas* were not tested for rural samples (Fig. 3). Isolation of co-pathogens with *V. cholerae* among the urban site has ETEC (7.15%) and *Campylobacter* (8.36%). But in the rural site *Campylobacter* isolated as a co-pathogen with *V. cholerae* was only 0.38%, whereas ETEC was not tested in the rural Matlab site for budgetary constraints (Supplementary table 2).

In the urban area, azithromycin susceptibility increased from 265 (8%) to 1,485 (48%) between 2006 and 2021; tetracycline susceptibility declined significantly (OR 0.74; 95% CI: 0.70, 0.77, *p *< 0.001) from 2,051 (45.9%) in 2000–2005 to 186 (4.2%) in 2011–2015 then again increased to half of its initial frequency 1,009 (22.6%). Similarly, ciprofloxacin susceptibility declined significantly from 2,581 (31.6%) in 2000–2005 to 1490 (18.2%) in 2016–2021 (OR 0.27; 95% CI: 0.17, 0.42, *p*-value < 0.001). Erythromycin susceptibility dropped substantially, from 2,155 (98%) to less than 21 (1%). In the rural area, a similar susceptibility trend and proportion were found for all the antibiotics. Doxycycline was only tested in the urban site from 2016 to 2021 and was 902 (100%) susceptible (Table 2).

**Table 2:** The Percentage of *V. cholerae* susceptible to tetracycline, erythromycin, ciprofloxacin, doxycycline, and azithromycin during the period, 2000–2021 (urban Dhaka and rural Matlab sites).

Years	Number of isolates tested	Azithromycin n = 3108 (%)	Doxycycline n = 902 (%)	Tetracycline n = 4467 (%)	Ciprofloxacin n = 8178 (%)	Erythromycin n = 2189 (%)
	**Urban site (Dhaka Hospital)**					
2000–05	2,582	**–**	**–**	2,051 (45.91)	2,581 (31.56)	2,155 (98.45)
2006–10	2,752	265 (8.53)	**–**	1,221 (27.33)	2,747 (33.59)	6 (0.27)
2011–15	1,370	1,358 (43.69)	**–**	186 (4.16)	1,360 (16.63)	7 (0.32)
2016–21	1,518	1,485 (47.78)	902 (100)	1,009 (22.59)	1,490 (18.22)	21 (0.96)
*OR (95% CI)		0.41 (0.12, 0.42)	**–**	0.74 (0.70, 0.77)	0.27 (0.17, 0.42)	0.01 (0.005, 0.009)
*P*-value		0.160	**–**	< 0.001	< 0.001	< 0.001

Years	Number of isolated tests	Azithromycin n = 439 (%)	Doxycycline (%)	Tetracycline n = 1,303(%)	Ciprofloxacin n = 2,111 (%)	Erythromycin n = 991 (%)
	**Rural site (Matlab Hospital)**					
2000–05	1,015	**–**	**–**	826 (63.39)	1,012 (47.94)	844 (85.17)
2006–10	562	**–**	**–**	251 (19.26)	556 (26.34)	136 (13.72)
2011–15	293	185 (42.14)	**–**	35 (2.69)	287 (13.60)	5 (0.50)
2016–21	259	254 (57.86)	**–**	191 (14.66)	256 (12.13)	6 (0.61)
*OR (95% CI)		**–**	**–**	0.60 (0.55, 0.66)	0.53 (0.27, 1.04)	0.08 (0.06, 0.09)
*P*-value		**–**	**–**	< 0.001	0.066	< 0.001

*OR* odds ratio; “**–**“: susceptibility pattern not checked for the antibiotics.

We analyzed the antibiotic susceptibility of *V. cholerae*, ETEC, and *Campylobacter* over the years. Ciprofloxacin showed 100% susceptibility against *V. cholerae* and ETEC, but with *Campylobacter* susceptibility decreased particularly in 2020 and 2021 (67.7%). There was also a decrease in tetracycline susceptibility for both ETEC (67.7%) and *Campylobacter* (80.6%) in 2021. *Campylobacter* showed decreased susceptibility for azithromycin (67.7%). The study also found an increasing number of ETEC as a co-pathogen with V. cholerae. Further analysis is needed to understand these findings better (Table 3).

**Table 3:** The antibiotic susceptibility trends in the co-pathogens along with *V. cholerae* (2009–2021).

	*V. cholerae* O1				ETEC					*Campylobacter*				
Year	Detection (%)	Ciprofloxacin	Azithromycin	Tetracycline	Detection (%)	Ciprofloxacin	Azithromycin	Tetracycline	ETEC**	Detection (%)	Ciprofloxacin	Azithromycin	Tetracycline	Campylobacter**
2009	600/2837 (21.1)	99.7	0	18.5	56/600 (9.3)	100	0	14.3	9.3	86/600 (14.3)	100	0	12.8	14.3
2010	514/2853 (18.0)	99.6	51.6	62.6	28/514 (5.4)	100	39.3	67.9	5.4	84/514 (16.3)	97.6	39.3	72.6	16.3
2011	251/2529 (9.9)	99.6	100	63.7	25/251 (10)	100	100	64	10	27/251 (10.8)	96.3	100	66.7	10.8
2012	331/2889 (11.5)	99.1	98.5	5.4	44/331 (13.3)	95	100	0	13.3	31/331 9.4)	90.3	90.3	12.9	9.4
2013	271/2303 (11.8)	99.3	99.3	1.5	20/271 (7.4)	100	95	0	7.4	30/271 (11.1)	96.7	96.7	3.3	11.1
2014	278/2957 (9.4)	98.9	98.9	0.7	24/278 (8.6)	100	100	0	8.6	49/278 (17.6)	95.9	95.9	4.1	17.6
2015	239/2784 (8.6)	99.6	99.2	0.8	20/239 (8.4)	100	100	0	8.4	35/239 (14.6)	100	97.1	0	14.6
2016	153/2737 (5.6)	98.7	98.7	1.3	24/153 (15.7)	100	100	0	15.7	21/153 (13.7)	95.2	100	4.8	13.7
2017	254/3058 (8.3)	100	99.6	0.4	25/254 (9.8)	100	100	0	9.8	45/254 (17.7)	100	100	0	17.7
2018	366/3353 (10.9)	100	98.6	76.2	72/366 (19.7)	98.6	98.6	79.2	19.7	76/366 (20.8)	100	98.7	73.7	20.8
2019	476/3917 (12.2)	100	99.2	98.1	63/476 (13.2)	100	98.4	98.9	13.2	93/476 (19.5)	98.9	98.9	98.9	19.5
2020	95/2030 (4.7)	100	90.5	98.9	14/78 (17.9)	100	92.6	82.4	17.9	17/95 (17.9)	82.4	76.5	100	17.9
2021	174/2753 (6.3)	100	93.1	95.4	32/174 (18.4)	100	96.9	67.7	18.4	31/174 (17.8)	67.7	67.7	80.6	17.8

**Detection rate as co-pathogen, unit = %.

In Table 4 we tried to illustrate the similar studies conducted from the icddr,b surveillance data set for the last 25 years using similar *V. cholerae* trends. We found only 5 studies where the author tried to show the changing trends of *V. cholerae* in urban/ rural populations except one where the author tried to show the urban and rural features. In our analysis, we have observed the changing pattern of *V. cholera* among different age groups and between urban and rural populations.

**Table 4:** Findings of articles on *V. cholera* trends from urban and rural Bangladesh.

Ref (pub. Yr.)	Primary Author	Data analyzed	Study site	Method of cholera detection	Strain	Co-pathogen detection	Antibiotic susceptibility check	Findings
(1996)	ASG Faruque	1992–1995	Urban (Dhaka Hospital)	Stool culture-based surveillance	*V.cholerae* 0139 And *Vibrio cholerae* O1	No	No	Cholera cases in 1993 were primarily caused by *V. cholerae* 0139, with *V. cholerae* 01 only accounting for a minor number of cases. *V. cholerae* 01 reemerged as the main cholera strain in the later part of the research period (Jan 1994–May 1995)
(2020)	K. Zaman	1974–2018	Rural (Matlab)	Stool culture-based surveillance	Cholera cases	No	No	Despite tendencies toward rising air and sea surface temperatures, which are known to enhance the incidence of cholera, and despite the persistently high cholera burden in many other regions of Bangladesh. In Matlab, cholera transmission has almost completely disappeared
(2002)	Ira M. Longini	1966–1998	Rural (Matlab)	Stool culture-based surveillance	Vibrio cholerae serogroup, serotype, and biotype	No	No	Between 1966 and 1988, both the classical and El Tor biotypes coexisted and lasted; the classical biotype disappeared by 1988, and the O139 serogroup first appeared in 1993. The Ogawa and Inaba serotypes were in constant circulation. Short-term population level immunity may be selectively conferred by Inaba for a longer time than by Ogawa
(2014)	Erik H. Klontz	2000–2012	Urban Dhaka and rural Matlab	Stool culture-based surveillance	*Vibrio cholerae* O1	No	Yes	The levels of erythromycin and tetracycline resistance suddenly rose in 2004–2005. At both study sites, resistance levels changed drastically and in a comparable way. Ciprofloxacin and azithromycin, which were introduced as a result of the sudden increase in tetracycline resistance, have demonstrated high susceptibility (> 99%) to *V. cholerae*
(2020)	Irin Parvin	2000–2018	Urban (Dhaka Hospital)	Stool culture-based surveillance	*Vibrio cholerae* O1	No	Yes	From 2000 through 2006, Vibrio cholerae O1 Inaba serotype predominated, from 2007 to 2015, the Ogawa serotype gradually gained dominance, b ut isolation rates fell to 1% in 2017, respectively, before rising to 75% in 2018. Between 2000 and 2004, almost all *V. cholerae* O1 strains were tetracycline sensitive. After that, it was found that the sensitivity was continuing to decline, reaching a low of 6% from 2012 to 2017 before rising once more to 76% in 2018. Throughout the course of the trial, susceptibility to ciprofloxacin and azithromycin was close to 100%, whereas susceptibility to cotrimoxazole was 1%

Supplementary table 3 shows the monthly count of isolated *V. cholerae* cases over the past 20 years for both urban and rural populations. The data indicates a consistent increase in the number of *V. cholerae O1* cases during April and May over the last two decades. Cholera dynamics in endemic regions display regular seasonal cycles at the beginning of summer and pronounced interannual variability. The mechanistic basis for a climate–cholera connection, which is likely to involve multiple pathways, remains poorly understood. The delta region of the Ganges and the Bramaputra has long been identified as cholera’s ‘native habitat’ and a source for the periodic pandemic spread of the disease. Despite huge population densities and poor WASH conditions in South-East Asia could not prevent cholera transmission, indicating that other local determinants are critical for cholera maintenance. But in 2020 probably due to covid-19 pandemic the number of cholera cases was the lowest (only 95), which may be the cause of maintaining hand hygiene and fewer hospital admission due to strict lockdown.

## Discussion

The analysis was conducted to describe the socio-demographic characteristics, co-pathogens, and antibiotic susceptibility of cholera patients in urban and rural Bangladesh between 2000 and 2021. In our study, the urban area had four times the number of cholera cases compared to the rural area. Studies reported in Bangladesh, cholera is gradually becoming an urban illness, particularly in Dhaka, the country's capital^19–21^. In the last two decades, Dhaka has also seen large-scale cholera epidemics, particularly amid major floods in 2007 and 2009^19^. Residents in Dhaka's urban slums are still at risk of contracting cholera^19^. Currently, the majority of cholera cases occur in the Asian region, and accurately assessing the disease's actual burden is challenging due to a lack of reporting or inaccurate reporting^7^.

According to the analysis of patient demographics in this study, the case distribution was roughly equal by sex, which is typical of past studies carried out in Bangladesh and other cholera-endemic nations^18,21^. Our findings showed that the majority of cholera-positive cases presented at both sites were 15 years and more. It is concerning that the proportion of cholera infections was steadily rising across all year groups. This is consistent with the results of earlier studies. In a previous study conducted at icddr,b Dhaka hospital, and other surveillance sites in Bangladesh, it was reported that older children and adults had greater diarrheal rates than those in the younger age group^20,22,23^. In Bangladesh, a national surveillance program for enteric diseases with laboratory confirmation was done in most regions of the country to understand the epidemiology of cholera^18^. The risk of cholera among patients with diarrhea younger than 5 years of age was lower than in other age groups, with those aged 5–17 years old having a 5.1 fold, those aged 18–45 years old having a 3.8 fold, and those aged 46 or older have a 2.7 fold increased risk of cholera in comparison with those under five^18^. Another study done in Nepal reported cholera infection was higher in males, of the adult age group (21–30 years)^24^. In all age groups, boiling water was found to be a significant protective factor. Contrarily, the waste disposal in the courtyard was a serious concern for disease transmission. Some researchers evaluated cholera risk depending on water treatment^25^. Cholera risk may be impacted by access to and usage of optimal hygiene measures^25^.

The preferred method of treating diarrheal disease is fluid and electrolyte replacement through oral hydration or intravenous (IV) fluid therapy. Even though the majority of episodes are self-limiting, however, there are some situations where antibiotic therapy is advised. Shigellosis, cholera, and campylobacteriosis are acute diarrheal disorders for which antimicrobial therapy is unquestionably effective^26^. Our study found that *Campylobacter* and ETEC were the top most co-pathogens. It is concerning that over the course of our 20-year study, the prevalence of *Campylobacter* increased by fourfold and that of ETEC increased by fivefold. A study conducted at icddr,b’s Dhaka Hospital between March 2019 and March 2020 with over five years of population yielded similar results^27^. The study reported that the two most common co-pathogens with *V. cholerae* were ETEC (12.79%) and *Campylobacter* (4.5%) in the research population^27^. However, we found considerably higher rates of ETEC (39.2%) and *Campylobacter* (40.7%) from 2016 to 2021 as co-pathogens. This finding was alarming in the urban site. However, we have no clear explanation for this finding. There might be a chance of a gradual decrease in azithromycin susceptibility which causes an increasing number of *Campylobacter* and ETEC along with *V. cholerae* as co-pathogens. Another observation was the presence of *Campylobacter* as co-pathogen in the urban site more than rural site. These open the window for further study in future.

A serious area of worry is the steadily rising antimicrobial resistance (AMR) among intestinal infections in patients, particularly in the critical care unit^26,28^. Controlling bacterial resistance requires knowledge, among the health workforce, of the susceptibility patterns of bacteria in various geographic areas^26^. In LMICs, antimicrobial resistance is incredibly poorly understood and monitored^26,28^. In our study, azithromycin susceptibility increased to 48%, however, erythromycin susceptibility decreased dramatically from 98% to below 1%. A study done at icddr,b found very high macrolide drug resistance to cholera pathogens. Additionally, they showed limited resistance of 1% to tetracycline and fluoroquinolone/quinolone drugs. We have also observed, that after a prolonged decline in both urban and rural sites, tetracycline and ciprofloxacin susceptibility was about to rise. Previously, Ahmed *et* al. reported high resistance to tetracycline and erythromycin from 2005 to 2008 in all age groups in Dhaka^26^. Local antibiotic susceptibility patterns ought to guide antibiotic selection. Doxycycline is advised as a first-line treatment for adults (including pregnant women) and children in the majority of nations according to CDC^29^. Currently, icddr,b is using doxycycline as a first-line drug for over 8 years of age based on local antibiotic susceptibility patterns.

Some innate characteristics of the patient were also related to cholera risk and seem to have enhanced natural selection in Bangladesh^30^. The likelihood of developing symptoms of cholera is enhanced by several hereditary traits, including genes in the Nuclear factor kappa B (NF-*k*β) pathway (NF-*k*β is an ancient protein transcription factor and considered a regulator of innate immunity^31^. The NF-* k*β signaling pathway links pathogenic signals and cellular danger signals thus organizing cellular resistance to invading pathogens)^27,31^, blood group “O”^25,30^, and the secretor status of individuals as reported in concurrent literature^32^. Cholera will undoubtedly continue to affect underprivileged groups who lack access to clean water, good hygiene habits, and sanitary facilities in the future^18^. To track development toward eradication and to determine the effect of measures like oral cholera vaccines (OCV), disease surveillance of environmental parameters that trigger outbreaks must be maintained and expanded in the coming years. Investing heavily in sustainable water and sanitation infrastructure is necessary for the long-term control of cholera. Especially in LMIC, timely and realistic projections are crucial to allow public health officials to plan and implement response measures.

Although, people in Dhaka are familiar with the icddr,b Diarrheal Hospital, a renowned “Cholera Hospital” with over 60 years of operation. Given that the icddr,b Hospital receives patients from a great distance and also serves as a the referral facility for diarrhea. Extensive hospital-based surveillance revealed that cholera was present in every Bangladeshi region that was being monitored^18^. This study employed a standard culture approach rather than PCR for the confirmation of cholera cases, PCR may increase sensitivity, particularly in the presence of antibiotics^33,34^. Data on clinical outcomes and mortality are also limited because the patients were not followed up after treatment or discharge. In the rural site, ETEC and *Aeromonas* were not tested for culture due to budgetary constraints, so for ETEC as a co-pathogen, we could not compare the urban and rural data, this is another limitation of our study. Climatic factors such as water temperature would drive seasonality through their direct influence on the abundance and/or toxicity of *V. cholerae* in the environment, however, our surveillance data has no provision for these data collection. We are planning to add those climate variables in our future study beside the population density and WASH conditions.

Cholera is an endemic disease in many areas of Bangladesh, and the illness recurs twice every year. Clinicians need access to up-to-date information on antimicrobial susceptibility and treating hospitalized patients. Controlling bacterial resistance requires knowledge among the health workforce about the susceptibility patterns of bacteria in various geographic areas. Finally, to achieve the WHO-backed objective of eliminating cholera in Bangladesh by 2030, a multi-sectoral approach that includes all ministries of the Bangladesh Government and international partners essentially needs to work together to improve water and sanitation practices, strengthen national health systems, and carry out OCV campaigns strategically.

## Methodology

### Ethics statement

We obtained informed consent from the participants or their parents or legal guardians before enrolling them in the study. The institutional review board (IRB; named Research Review Committee and Ethical Review Committee) of icddr,b reviewed and approved the study protocol and the consenting procedure as all the information collected was compatible with the information that is needed for quality patient care. All methods were performed in accordance with the relevant guidelines and regulations.

### Study design

The study used data from facility-based diarrheal disease surveillance systems (DDSS) that monitored patients who sought care for diarrheal diseases in the urban Dhaka Hospital and the rural Matlab Hospital of icddr,b^35^. Patients from Dhaka Hospital were from the urban and peri-urban areas of Dhaka city. Patients from Matlab Hospital were from the villages that are under the census process of Matlab Health and Demographic Surveillance System (HDSS) of icddr,b^36^.

These specialized research and training health facilities primarily provide care to patients with diarrheal illnesses or acute respiratory infections with or without associated complications and health problems. Most of the patients are from poor socioeconomic strata. Since its inception, these facilities provide free-of-cost care to the patients and, for the last 10 years, 140,000–190,000 patients annually were treated in the Dhaka Hospital. 18,000–48,000 patients were treated annually at the Matlab Hospital, but only about 3–7% of them are from the census population of HDSS.

The Dhaka Hospital, since its inception in 1979, icddr,b had been operating DDSS that systematically samples patients to collect demographic, socioeconomic, and clinical information using a standard structured questionnaire. This included a 4% sample from 1979 through 1995; then a 2% sample from 1996. At the Matlab Hospital, data from patients seeking care were recorded in the surveillance system since 1999, if they live in villages as defined in this area. In 2014, the population of the 142 villages in the HDSS was 230,185^36^. Demographic, socioeconomic, clinical, and laboratory data collected from all patients enrolled in the DDSS of Dhaka Hospital and Matlab Hospital of icddr,b between January 2000 to December 2021 were utilized to form the analyzable dataset. Data were included from diarrheal patients (three or more loose stools per 24 h) of all age groups who were treated in the Dhaka Hospital of icddr,b and enrolled in the DDSS, or diarrheal patients who were treated at the Matlab Hospital and resident of the villages belonging to the HDSS during the study period.

### Laboratory methods

Fresh stool specimens collected from all enrolled patients were immediately transported to the clinical microbiology laboratories of the respective hospitals, following an appropriate cold chain. In the Dhaka Hospital, stool samples were routinely screened for common enteric pathogens including *V. cholerae*, *Enterotoxigenic Escherichia coli* (ETEC), *Shigella* spp., *Salmonella* spp., and Rotavirus^37^ from stool culture using pathogen-specific isolation, inoculation, incubation, and subculture standard laboratory technique. The stool was cultured on tellurite taurocholate gelatin agar (TTGA) media for *V. cholerae.* Specimens were inoculated for enrichment on alkaline bile peptone broth and TTGA and incubated overnight. Colony identification was performed after plating on TTGA^38^. At the Matlab Hospital, laboratory diagnoses were carried out to isolate *V. cholerae, Salmonella* spp. *Shigella* spp. and Rotavirus isolation, identification, sero-grouping, and bio-typing of these bacterial pathogens were performed using standard laboratory procedures^37^. Antimicrobial susceptibility was determined by the standard disc diffusion method on Muller-Hinton agar with commercial discs (BD, Becton, Dickinson, and Company, USA) and the interpretative categories of sensitive, intermediate, and resistant were determined based on the cutoff of the zone size for antibiotics according to the up-to-date Clinical and Laboratory Standards Institute guidelines for *V. cholerae* (Weinstein MP. Clinical Laboratory Standards Institute. Performance Standards for Antimicrobial Disk Susceptibility Tests; Approved Standard. CLSI Document M2–A9. Wayne, PA: Clinical Laboratory Standards Institute; 2018.)^39^.

Of the stool specimens tested for selected conventional etiologies in last 20 years (*V. cholerae*, *Shigella*, *Salmonella*, ETEC, *Campylobacter* and Rotavirus) 50% were negative for these enteric pathogens. And of the 8222 conventional culture-confirmed *V. cholerae* O1 all were of El Tor biotype, those represented Inaba [n = 3022; (37%) and Ogawa (n = 5198; 63%)] serotypes.

### Data analysis

We reported the patients' clinical, household, and sociodemographic characteristics by using frequency as a percentage for categorical variables to summarize the data. The households were categorized into five socioeconomic status (SES) groups: poor, lower-middle, middle, upper-middle, and rich, using principal component analysis^40^. Variables we used to construct the asset index were ownership of radio/cassette player, television, electric fan, cot, almirah (cabinet), floor, roof, and wall material of the house, and the type of fuel used for cooking. Individuals were sorted based on their asset index score, and cutoff values for the percentiles of the population were established. The households were then assigned to a group based on their asset index score, with the bottom 20% referred to as "poorest," the next 20% as "lower middle," the following 20% as "middle," the subsequent 20% as "upper middle," and the top 20% as "richest." However, it is important to note that this classification does not adhere to the conventional poverty definitions^41^. To assess the association between the presence of *V. cholerae* in stool and the change during a 5-year interval from 2000 to 2021, multiple logistic regression was used on several outcome variables within a year of patient admission (different period: 2000–2005, 2006–10, 2011–15, and 2016–2021) where 2000–2005 served as the reference group and adjusted for patients age, sex, the status of breastfeeding (under-3 children), use of antibiotic before hospitalization, number of family members, parental education, drinking water (water sources in the study: tape water, pond/river, and tube well water. For ease of analysis, we grouped them into two categories: tube well water and non-tube well water (included tape and pond/river water)), toilet facility, water treatment method (boiling and no treatment), garbage disposal method, asset index and urban and rural sites suggesting the association with the outcome as indicated in the literature. The general equation used for MLR is: ln[Y/(1 − Y)] = a + b1X1 + b2X2 + b3X3… we found the slopes (b1, b2, etc.) and intercept (a) of the best-fitting equation in a multiple logistic regression using the maximum-likelihood method, rather than the least-squares method used for multiple linear regression. The variance inflation factor (VIF) was calculated to detect multicollinearity, and no variable with a VIF value greater than 5 was identified in the final model. We estimated the coefficient and its 95% CI to describe the precision of the point estimate. During the analysis, a *P* value of < 0.05 was considered statistically significant. STATA 17.0 IC (Stata Corp LLC, College Station, TX) was used to analyze the entire data set.

## Supplementary Information

Supplementary Information.

## Data Availability

The dataset analyzed during the current study is not publicly available due to it is prudent to mention that the data of this manuscript has been obtained from a large hospital surveillance with a huge data set. This data set contains some personal information of the study patients (such as name, admission date, month, area of residence). However, during taking the consent from the patients, it has been ensured that the personal information of them will not be disclosed, but, the study results will be published. Thus, the availability of this whole data set in the manuscript, the supplemental files, or a public repository will open all the personal information of the patients that should not be disclosed; additionally, this will disclose other important information those are yet to be published. Thus, the policy of icddr,b is that we should not make the availability of the whole data sets in the manuscript, the supplemental files, or a public repository. However, part of the data set related to this manuscript is available upon request, and readers may contact Ms. Armana Ahmed (aahmed@icddrb.org) of the Research Administration & Strategy of icddr,b to request the data (http://www.icddrb.org/).
