# Supplementary material for: Vibrio cholerae in rural and urban Bangladesh, findings from hospital-based surveillance, 2000–2021
Source: Sci Rep. 2023 Apr 19;13:6411. doi: 10.1038/s41598-023-33576-3 (PMC10115832; doi:10.1038/s41598-023-33576-3)
Supplement: Supplementary file 1 — Supplementary Information. [file 41598_2023_33576_MOESM1_ESM.docx]

**Supplementary table 1.** General characteristic of the *V. cholerae* positive diarrhea patients admitted in 2000-2021 in icddr,b Dhaka Hospital (urban) and Matlab Hospital (rural), Bangladesh

| **Characteristics** | | **Urban** | **Rural** |
| --- | --- | --- | --- |
|  |  | *n=8,221* | *n= 2,129* |
| **Age group** | |  |  |
|  | <5y | 1,642(19.97) | 483(22.69) |
|  | 5-15y | 1,343(16.34) | 438(20.57) |
|  | 15-60y | 4,874(59.29) | 1,016(47.72) |
|  | >60y | 362 (4.40) | 192 (9.02) |
| **Sex (**female) | | 3,554(43.23) | 1,099(51.62) |
| **Anthropometry** (under 5 children) | |  |  |
|  | Stunted | 655(41.59) | 230(48.32) |
|  | Wasted | 572(36.48) | 193(40.72) |
|  | Underweight | 841(53.16) | 292(61.09) |
| **Breastfeeding status** (0-36 months) | | *n=1629* | *n=473* |
|  | Non-breastfed | 770 (47.27) | 248 (52.73) |
|  | Breastfed | 859 (52.43) | 225 (47.57) |
| **Asset index** | |  |  |
|  | Poor | 1,655 (20.13) | 851 (40.07) |
|  | Lower middle | 354 (4.31) | 940 (44.26) |
|  | Middle | 3,170 (38.56) | 205 (9.65) |
|  | Upper middle | 1,054 (12.82) | 32 (1.51) |
|  | Rich | 1,987 (24.17) | 96 (4.52) |
| **More than 5 family members** | |  |  |
|  | Yes | 4,471 (54.39) | 1,478 (69.45) |
| **Paternal education** (under 5 children) | | *n=1,640* | *n=483* |
|  | Illiterate | 623 (37.99) | 150 (31.06) |
|  | Up to Primary | 380 (23.17) | 145 (30.02) |
|  | Above primary | 637 (38.84) | 188 (38.92) |
| **Maternal education** (under 5 children) | | *n=1,640* | *n=483* |
|  | Illiterate | 611 (37.26) | 137 (28.36) |
|  | Up to Primary | 405 (24.70) | 131 (27.12) |
|  | Above Primary | 624 (38.05) | 215 (44.51) |
| **Patients education** (>15y) | |  |  |
|  | Illiterate | 1,842 (22.41) | 386 (18.14) |
|  | Up to Primary | 1,363 (16.58) | 368 (17.29) |
|  | Above Primary | 2,168 (26.37) | 507 (23.83) |
|  | Not applicable | 2,847 (34.64) | 867 (40.74) |
| **Toilet facility** | |  |  |
|  | Sanitary | 173 (2.1) | 27 (1.27) |
|  | Semi sanitary | 5,587 (67.97) | 233(10.95) |
|  | Non-sanitary/ others | 2,460 (29.93) | 1,868 (87.78) |
| **Water treatment method** | |  |  |
|  | Boil | 5,455 (69.04) | 2,014 (98.63) |
|  | No treatment | 2,446 (30.96) | 28 (1.37) |
| **Disposal of garbage** | |  |  |
|  | Outside | 7,510 (91.45) | 2,112 (99.44) |
|  | courtyard | 702 (8.55) | 12 (0.56) |
| **Dehydration status** | |  |  |
|  | No dehydration | 517(6.29) | 398 (18.7) |
|  | some/ severe dehydration | 7,697 (93.71) | 1,730 (81.3) |
| **Rehydration fluid required** | |  |  |
|  | None | 71 (0.86) | 6 (0.28) |
|  | ORS only | 1,708 (20.78) | 781 (36.68) |
|  | IV fluid only | 56 (0.68) | 36 (1.69) |
|  | ORS and IV fluid | 6,386 (77.68) | 1,306 (61.34) |

IV fluid: Intravenous fluid; Stunting: height/length for age z score <-2; Wasting: weight for height z score <-2; Underweight: weight for age z score <-2

**Supplementary table 2**. Multiple pathogen isolated along with *V. cholerae* (+) patients admitted during 2000-2021

| **Multiple pathogen** | Dhaka **n (%)** | Matlab **n (%)** |
| --- | --- | --- |
| *V. cholerae* + enterotoxigenic *E. coli* | 587 (7.15) | - |
| *V. cholerae* + *Aeromonas* spp. | 10 (0.12) | - |
| *V. cholerae* + *Campylobacter* spp. | 687 (8.36) | 8 (0.38) |
| *V. cholerae +* *Salmonella* spp. | 37 (0.45) | 22 (1.03) |
| *V. cholerae* *+* *Shigella* spp. | 126 (1.53) | 35 (1.64) |
| *V. cholerae* + Rotavirus | 285 (3.47) | 60 (2.82) |

“- “: no organism isolated

**Supplementary Table 3.** Monthly isolation of V. cholerae O1 in Dhaka and Matlab Hospital (2001-2021)

|  |  | ***V. cholerae O1*** | | | | | | | | | | | | | | | | | | | | |
| --- | --- | --- | --- | --- | --- | --- | --- | --- | --- | --- | --- | --- | --- | --- | --- | --- | --- | --- | --- | --- | --- | --- |
| **Month** | **Overall** | **2001** | **2002** | **2003** | **2004** | **2005** | **2006** | **2007** | **2008** | **2009** | **2010** | **2011** | **2012** | **2013** | **2014** | **2015** | **2016** | **2017** | **2018** | **2019** | **2020** | **2021** |
| **January** | **205** | 11 | 19 | 12 | 17 | 18 | 8 | 6 | 4 | 14 | 12 | this | 13 | 7 | 2 | 11 | 5 | 7 | 1 | 6 | 15 | 11 |
| **February** | **173** | 4 | 20 | 6 | 4 | 13 | 10 | 4 | 5 | 4 | 17 | 5 | 9 | 0 | 1 | 5 | 0 | 9 | 10 | 9 | 16 | 16 |
| **March** | **631** | 4 | 13 | 9 | 28 | 54 | 34 | 18 | 6 | 79 | 100 | 24 | 63 | 11 | 16 | 21 | 10 | 16 | 29 | 34 | 9 | 43 |
| **April** | **1,390** | 57 | 41 | 44 | 73 | 144 | 107 | 43 | 36 | 142 | 143 | 26 | 99 | 25 | 44 | 52 | 20 | 39 | 113 | 86 | 1 | 12 |
| **May** | **1,042** | 46 | 35 | 62 | 60 | 88 | 83 | 44 | 84 | 56 | 40 | 23 | 64 | 33 | 44 | 40 | 19 | 42 | 76 | 53 | 0 | 7 |
| **June** | **668** | 21 | 25 | 42 | 47 | 39 | 42 | 85 | 32 | 44 | 17 | 24 | 26 | 47 | 33 | 25 | 15 | 12 | 27 | 12 | 2 | 11 |
| **July** | **540** | 12 | 32 | 18 | 51 | 33 | 16 | 78 | 41 | 33 | 20 | 21 | 15 | 31 | 27 | 19 | 5 | 9 | 13 | 30 | 0 | 6 |
| **August** | **763** | 6 | 40 | 20 | 57 | 64 | 41 | 155 | 45 | 88 | 29 | 32 | 4 | 11 | 8 | 11 | 25 | 37 | 34 | 30 | 3 | 2 |
| **September** | **838** | 5 | 30 | 26 | 106 | 86 | 117 | 63 | 72 | 39 | 13 | 9 | 8 | 46 | 47 | 20 | 10 | 11 | 15 | 64 | 0 | 4 |
| **October** | **940** | 26 | 26 | 57 | 91 | 80 | 119 | 57 | 24 | 32 | 68 | 43 | 17 | 49 | 31 | 11 | 18 | 27 | 26 | 67 | 19 | 8 |
| **November** | **624** | 21 | 48 | 39 | 37 | 32 | 22 | 52 | 24 | 52 | 42 | 28 | 6 | 8 | 17 | 17 | 15 | 34 | 17 | 44 | 15 | 23 |
| **December** | **408** | 21 | 33 | 19 | 36 | 33 | 18 | 27 | 16 | 17 | 13 | 13 | 7 | 3 | 8 | 7 | 11 | 11 | 5 | 41 | 15 | 31 |
| **Total** | **8,222** | **234** | **362** | **354** | **607** | **684** | **617** | **632** | **389** | **600** | **514** | **251** | **331** | **271** | **278** | **239** | **153** | **254** | **366** | **476** | **95** | **174** |
| Per Month | 685 | 20 | 30 | 30 | 51 | 57 | 51 | 53 | 32 | 50 | 43 | 21 | 28 | 23 | 23 | 20 | 13 | 21 | 31 | 40 | 8 | 15 |
